# Effect of WeChat‐based continuous care intervention on the somatic function, depression, anxiety, social function and cognitive function for cancer patients: Meta‐analysis of 18 RCTs

**DOI:** 10.1002/nop2.1916

**Published:** 2023-06-26

**Authors:** Zhuoxin Wang, Shanshan Deng, Hekai Lv, Yanyan Fan, Li Zhang, Fuzhi Wang

**Affiliations:** ^1^ School of Nursing Bengbu Medical College Bengbu China; ^2^ The Second People's Hospital of Changzhi Changzhi Shanxi Province China; ^3^ School of Health Management Bengbu Medical College Bengbu China; ^4^ School of Public Health Peking University Beijing China

**Keywords:** cancer, continuous care, meta‐analysis, WeChat

## Abstract

**Aim:**

This meta‐analysis systematically reviewed and identified the effects of WeChat‐based continuous care (WCC) interventions on various outcomes in cancer patients.

**Design:**

Systematic review and meta‐analysis.

**Methods:**

In this study, outcome measures included somatic function, anxiety, depression, social function, and cognitive function. The standardized mean differences and 95% CIs of pooled effect sizes were calculated using fixed‐ and random‐effects models. N_Fail‐safe_ and Begg's tests were performed to evaluate publication bias, and sensitivity analysis was performed to evaluate the robustness of the meta‐analysis results.

**Results:**

The meta‐analysis included 18 RCTs of moderate quality. WCC interventions significantly improved somatic function, depression, anxiety, social function, and cognitive function in cancer patients. There was no significant publication bias, and the sensitivity analysis indicated robust results.

**Patient or Public Contribution:**

WCC interventions improved depression, anxiety, social function, and cognitive function in cancer patients.

## 1 INTRODUCTION

Cancer is a malignant disease characterized by high mortality; it is difficult to treat and places a heavy burden on patients and their families (Cao et al., 2020). According to the 2020 Global Cancer Annual Report, there were approximately 19.3 million new cancer cases and 10 million cancer deaths worldwide in 2020, representing a significant cause of morbidity and mortality (Sung et al., 2021). In China, there were approximately 4.57 million new cancer cases in 2020, accounting for approximately 24% of new cases worldwide, and 3 million deaths, which accounted for approximately 30% of global cancer‐related deaths (Cao et al., 2021).

To alleviate the economic burden caused by the high incidence and mortality of cancer, the concept of tertiary prevention has been proposed in China. Third‐level prevention refers to measures such as preventing recurrence, reducing complications, preventing disability, improving survival and recovery rates, relieving pain caused by cancer, improving quality of life, and promoting rehabilitation for patients with existing cancer (Maomao & Wanqing, 2021). Continuous care is a new model of care that has emerged in response to social developments and changes in healthcare services. This involves inpatient care that extends to the treatment and rehabilitation of patients after discharge, helping to improve patient self‐management and ensuring uninterrupted information, treatment and care services (Hirschman et al., 2015; Liu et al., 2015). For patients with cancer, reasonable out‐of‐hospital health guidance can, to a certain extent, change poor health behaviours, reduce the likelihood of hospital readmission (Chen et al., 2019), and reduce the mortality rate of cancer patients (Justiniano et al., 2019), which is one of the important measures of tertiary cancer prevention. Compared to routine discharge follow‐up care, continuous care can prevent postoperative cancer fatigue (Huanzhi et al., 2017) and reduce anxiety and depression in cancer patients (Yingying et al., 2019).

There are an increasing number of forms of continuous care for cancer patients, such as the establishment of nursing clinics and continuity of care centres, telephone follow‐up visits, and the creation of online platforms (Chen et al., 2019; Ye et al., 2016). However, there are still a series of problems with regard to its development, such as insufficient human resources, imperfect coordination mechanisms between hospitals and communities (Li & Yimin, 2018), insufficient awareness of patients about continuity of care, and low cooperation (Easley et al., 2016). With rapid economic development, mobile applications have started to be widely used in the health field (Higgins, 2016), such as storing patients' medical records and providing online health information, appointment reminders, and telemedicine (Lewis et al., 2016). WeChat, an application launched by Tencent in 2011, has become one of the most popular social software programs in China, and many researchers are exploring its application in the field of continuous care (Jingjing et al., 2016). The application provides voice, text and image sharing among a large number of active users, with 1.225 billion active users as of 30 December 2020, and 5.2% growth year‐over‐year (Tencent website, 2021). Compared to traditional interventions, when using WeChat for interventions, patients are not only able to receive relevant health guidance in a timely manner (Ma et al., 2018) but also have voice communication and video consultation with medical staff (Lyu et al., 2016); this is an intervention method that addresses the spatial and temporal limitations of medical services and enables medical staff to provide specific medical aspects of health guidance to patients during follow‐up visits (Ma et al., 2018).

In recent years, the application of WeChat‐based continuous care (WCC) interventions in cancer patients has gradually developed, and a large number of experimental studies have been conducted by relevant researchers evaluating somatic, mental, cognitive, and social functioning (Zhao et al., 2020; Zhang Dongfang et al., 2019; Jiaoyan et al., 2020), but these studies have been small in scope and have reported inconsistent or even contradictory findings. A typical example was the study of improving depression in cancer patients. One study (Yu, 2017) reported a significant improvement (*p* < 0.001), but the improvement effect in another study was not particularly significant, *p* = 0.05 (Jiaoyan et al., 2020). A similar situation could be found in studies on somatic function. One study reported that WCC intervention was ineffective for somatic health (Xianghua et al., 2017), but another study (Lihui et al., 2018) showed a significant improvement in somatic health. The results of the current literature search indicated that there are no uniform standards and no standardized processes for the timing, periodicity, methods, content, and processes of WCC interventions, the implementation has varied greatly across locations, and there is a lack of a comprehensive evaluation of the effects reported in the literature. Therefore, the application of meta‐analysis to explore the effects of WCC interventions applied to cancer patients was studied, and the effectiveness of the interventions was quantitatively analysed, which has positive implications for the development of relevant clinical practice in the future.

## 2 MATERIALS AND METHODS

### 2.1 Literature search

We searched for randomized controlled trials (RCTs) published in two English databases (PubMed and Web of Science [WOS]) and 2 Chinese databases (China Biology Medicine [CBM] and China National Knowledge Infrastructure [CNKI]) in the last decade that involved the evaluation of the effectiveness of WCC interventions on somatic function, depression, anxiety, social function and cognitive function in cancer patients.

The search strategy was as follows: (1) WeChat, (2) continuity of patient care, (3) continuity of care, (4) continuing care, (5) continuous care, (6) transitional care, (7) extended care, (8) 2 or 3 or 4 or 5 or 6 or 7, (9) cancer, (10) oncology, (11) 9 or 10, (12) somatic function, (13) anxiety, (14) depression, (15) social function, (16) cognitive function, (17) 12 or 13 or 14 or 15 or 16, and (18) 1 and 8 and 11 and 17. The search date ended on Dec 31, 2020.

### 2.2 Selection procedure

A study was included in the present analysis if (a) the participants were cancer patients; (b) the intervention involved WCC; (c) the outcomes included somatic function, anxiety, depression, social function, or cognitive function; and (d) the study was an RCT. If the study included multiple assessment time points, data from the post‐intervention time point were chosen for the analysis. A study was excluded from the present study if (a) the articles received a poor quality evaluation (Physiotherapy Evidence Database scale [PEDro] score <4) or (b) the data for the study were incomplete or unavailable.

Two researchers independently searched and screened the literature in strict accordance with the inclusion and exclusion criteria and then extracted and cross‐checked the data, which included author, date of publication, country, sample size, age, gender, interventions, and outcome indicators of the included subjects. A third researcher was consulted to resolve any disagreement in the screening results.

### 2.3 Quality assessment

The PEDro scale (Moseley et al., 2019) was used to evaluate the effect of WCC interventions on cancer patients in 18 randomized clinical trials. PEDro was introduced by the Centre for Evidence‐Based Physiotherapy at The George Institute for Global Health. Eighteen studies with PEDro scores of less than 4, 4 to 5, 6 to 8, or 9 to 11 were considered to have poor, fair, good, or excellent methodological quality, respectively.

### 2.4 Computing effect sizes

For the meta‐analyses, the mean change from the baseline to post‐intervention assessment for the intervention and control groups was calculated as the effect size (ES). The group mean deviation and the pooled SD were calculated (Cochrane Handbook 16.1.3.2, The Cochrane Collaboration). When 2 or more intervention groups were included, the ES for only the most active group was calculated. Given that these variables are continuous outcome measures with different units of measurement, standardized mean differences (SMDs) were estimated using fixed‐ or random‐effects models with 95% confidence intervals (CIs).

### 2.5 Heterogeneity

Heterogeneity was explored using heterogeneity measures *χ* ^2^ and *I* ^2^. *I* ^2^ is the proportion of total variation observed between studies that are attributable to differences between studies rather than sampling error (chance). When *I* ^2^ > 50%, the studies in the meta‐analysis were considered to be heterogeneous, and a random‐effects model was used (Moazzami et al., 2020). Otherwise, a fixed‐effects model was used.

Sensitivity analyses were conducted to identify potential sources of heterogeneity and to determine how sensitive the final study conclusions were to a particular method or study design feature that was used (Thabane et al., 2013). If the effect and CIs in the sensitivity analyses led to the same conclusion as the primary meta‐analysis value, the results were considered robust.

### 2.6 Publication bias

The Begg test and fail‐safe number (NFS) were used to determine whether there was publication bias (Begg & Mazumdar, 1994; Gjerdevik & Heuch, 2014). The Begg test provides a statistical parameter for the evaluation of publication bias. If *Z* > 1.96, *p* < 0.05 indicates that publication bias may exist. Values of *Z* < 1.96 and *p* > 0.05 were considered to indicate no publication bias. NFS is a method of sensitivity analysis that calculates how many reports of negative results are needed to reverse the results when the analysis results are statistically significant (Hongyan, 2007). The larger the safety factor is, the more stable the meta‐analysis results. A reasonable level was achieved if the NFS exceeded 5*K* + 10 (where *K* is the number of studies in the meta‐analyses).

### 2.7 Ethics approval

This study was approved by the Ethics Committee of the Bengbu Medical College (2017054).

## 3 RESULTS

### 3.1 Study selection

**FIGURE 1:**
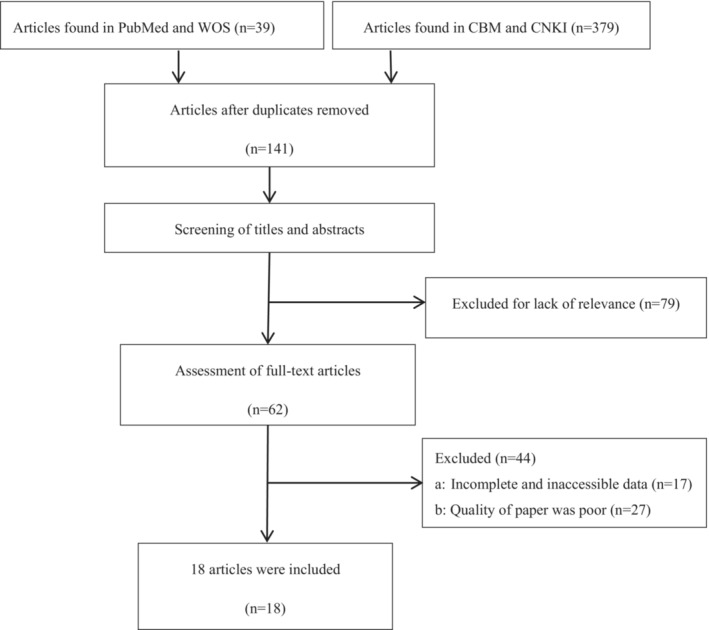
Flow diagram of study selection.

A total of 418 articles were retrieved from the 4 electronic databases (PubMed, WOS, CNKI and CBM). Of these, 277 duplicate articles were excluded, 79 articles were excluded because they were not relevant to our study, and 44 articles were excluded because they did not meet the inclusion criteria. Thus, 18 RCTs were included in this study, and there were no significant differences between the general information of patients in the experimental and control groups in each study. A total of 1622 cancer patients were included in the 18 studies, including 815 in the experimental group and 807 in the control group. The article selection process is described in the flow diagram (Figure 1).

### 3.2 Intervention programmes

There are three types of WCC interventions: (1) mixed interventions (including the delivery of cancer‐related knowledge through WeChat groups or WeChat public numbers and interactive communication between medical and nursing staff and cancer patients in WeChat groups) (Dongfang et al., 2019; Guolian, 2017; Hong, 2015; Lihui et al., 2018; Long et al., 2018; Shuangni et al., 2019; Xiuhong et al., 2020); (2) mixed interventions and psychological interventions (including the delivery of cancer‐related knowledge through WeChat groups or WeChat public numbers, interactive communication between medical and nursing staff and cancer patients in WeChat groups, and psychological guidance for patients with anxiety and depression) (Chen & Wang, 2016; Jing & Yajuan, 2016; Xiaoyu & Kuiling, 2017; Xianghua et al., 2017; Zhanhua, 2018; Ying & Fang, 2020); and (3) mixed interventions and rehabilitation exercises (including the delivery of cancer‐related knowledge through WeChat groups or WeChat public numbers, interactive communication between medical and nursing staff and cancer patients in WeChat groups, and guidance for cancer patients to perform functional exercises) (Lihui et al., 2018; Min et al., 2018; Sufang & Xiaoya, 2019; Xiaoyu et al., 2017; Xiuhong et al., 2020).

All interventions were delivered to cancer patients and were conducted by trained health care providers through WeChat groups or WeChat public accounts. The intervention durations ranged from 3 weeks to 12 months. The patients in the control groups were offered the usual discharge follow‐up intervention (Table 1).

**TABLE 1:** Study characteristics.

Author	Cancer	Sample size (IG/CG)	Intervention method		Follow‐up	Outcomes					PEDroScore
			IG	CG		Somatic function	Depression	Anxiety	Social function	Cognition function	
Song Hong 2015	Rectal CA	50/50	1	4	6 months				PAIS‐SR		8
Pan Jing 2016	Rectal CA	55/55	2	4	12 months	SF‐36			SF‐36		7
Chen Jie 2016	Cardiac CA	45/38	2	4	3 months		SDS	SAS	HHI		6
Yan Xiaoyu 2017	Laryngeal CA	50/50	2	4	6 months		SDS				6
Xu Xianghua 2017	Laryngeal CA	42/42	2	4	3 months	FACT‐H&N	SDS	SAS			8
Li Yu 2017	Breast cancer	40/40	3	4	3 months		HAMD	HAMA			6
Li Guolian 2017	Breast cancer	72/72	1	4	6 months	FACT‐B			FACT‐B		6
Jia Zhanhua 2018	Gastric CA	45/45	2	4	12 months	EQRTC QLQ‐C30	HAMD	HAMA	EQRTC QLQ‐C30	EQRTC QLQ‐C30	7
Ji Lihui 2018	non‐Hodgkin's lymphoma	40/39	1	4	6 months	EQRTC QLQ‐C30			EQRTC QLQ‐C30	EQRTC QLQ‐C30	6
Ling Long 2018	Breast CA	50/50	1	4	3 months				FACT‐B		8
Liu Min 2018	Lung CA	40/40	3	4	3 weeks		SDS	SAS			7
Wang Shuangni 2019	Cancer pain	42/42	1	4	4 weeks			SAS			7
Wei Sufang 2019	Breast CA	55/55	3	4	3 months	EQRTC QLQ‐C30			EQRTC QLQ‐C30	EQRTC QLQ‐C30	8
Zhang Dongfang 2019	Cancer pain	48/48	1	4	None		SDS	SAS			8
Li Qianqian 2020	Breast CA	37/37	3	4	6 months	FACT‐B			FACT‐B		6
Shen Xiuhong 2020	Lung CA	30/30	1	4	9 weeks			SAS			8
Zhang Ying 2020	Rectal CA	40/40	2	4	None	COH‐QOL‐OQ			COH‐QOL‐OQ		8
Cao Jiaoyan 2020	Prostatic CA	34/34	3	4	3 months	EQRTC QLQ‐C30	SDS	SAS	EQRTC QLQ‐C30	EQRTC QLQ‐C30	7

*Note*: 1, Mixed interventions; 2, Mixed interventions and psychological interventions; 3, Mixed interventions and rehabilitation exercises; 4, Routine discharge follow‐up (telephone follow‐up, regular return to hospital for checkups).

Abbreviations: CA, cancer; CG, control group; COH‐QOL‐OQ, City of Hope Quality of Life‐Ostomy Questionnaire; EQRTC QLQ‐C30, European organization for research and treatment of cancer quality life questionnaire core 30; FACT‐B, Functional Assessment of Cancer Therapy‐Breast; FACT‐H&N, Functional Assessment of Cancer Therapy‐Head and Neck; HAMD, Hamilton Anxiety Scale; HDMD, Hamilton Depression Scale; HHI, Hert Hope Index; IG, interventions group; PAIS‐SR, Psychosocial Adjustment to Illness Scale; SDS, Self‐rating Depression Scale; SAS, Self‐rating Anxiety Scale; SF‐36, Physical Function and Mental Health Scale of the Short Form‐36 Health Survey.

### 3.3 Outcome analyses

#### 3.3.1 Somatic function

**FIGURE 2:**
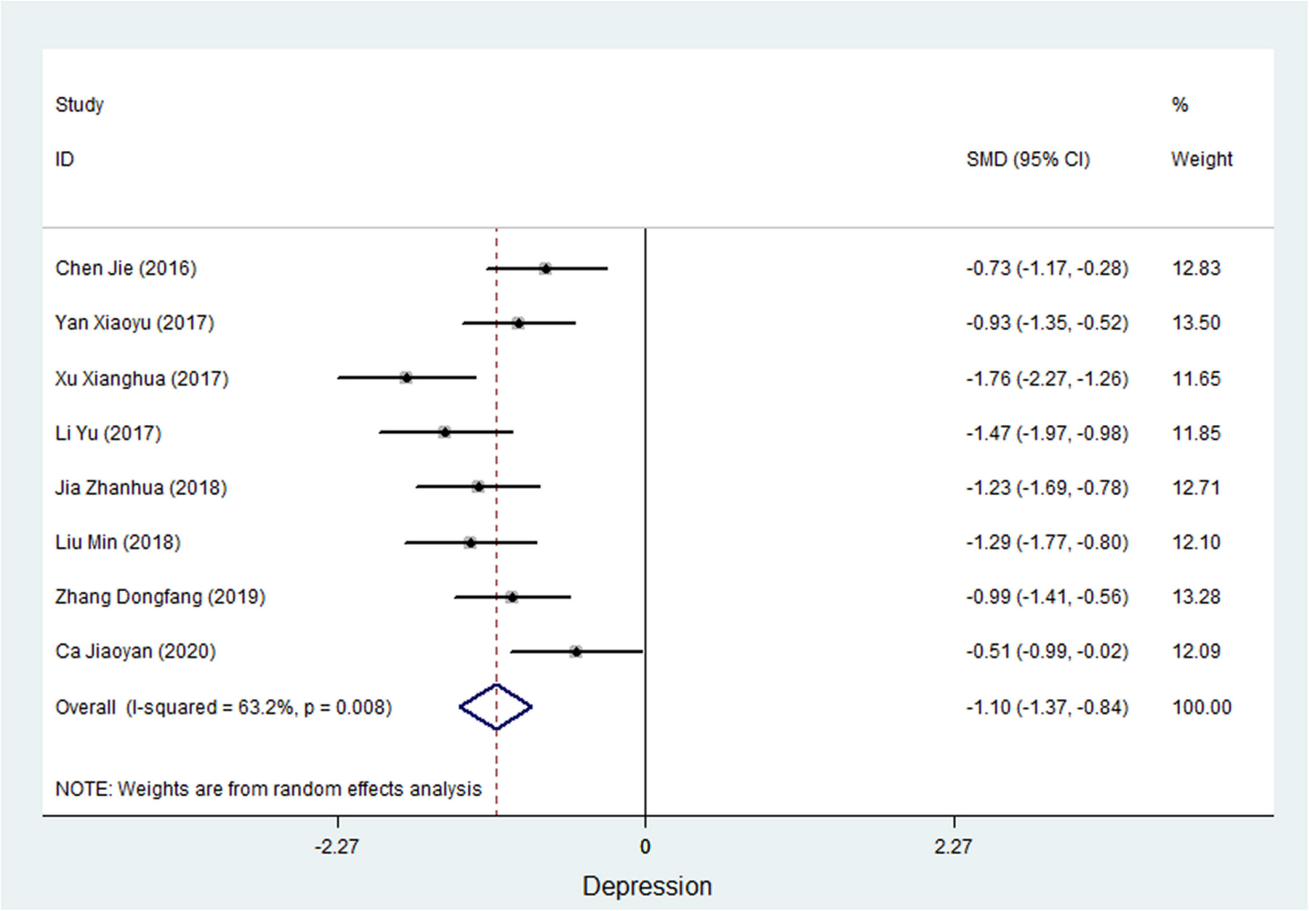
A forest plot of the ES of WCC on somatic functioning in cancer patients.

Nine studies measured the somatic function of cancer patients (Guolian, 2017; Jiaoyan et al., 2020; Jing & Yajuan, 2016; Lihui et al., 2018; Qianqian, 2020; Sufang & Xiaoya, 2019; Xianghua et al., 2017; Ying & Fang, 2020; Zhanhua, 2018). Of the 9 studies, 4 studies used the European Organization for Research and Treatment of Cancer quality of life questionnaire (EQRTC QLQ‐C30) to evaluate somatic function in patients, 2 studies used the Functional Assessment of Cancer Therapy–Breast Cancer (FACT‐B), 1 study used the 36‐item Short Form Survey (SF‐36), 1 study used the City of Hope‐Quality of Life‐Ostomy Questionnaire (COH‐QOL‐OQ), and 1 study used the Functional Assessment of Cancer Therapy–Head and Neck (FACT‐H&N) scale. Meta‐analysis showed that the heterogeneity test resulted in *I* ^2^ = 95.1%, and the SMD of the combined ES was 1.348 (95% CI, 0.653 to 2.043; *p* < 0.001) using a random‐effects model, suggesting that WCC improves somatic functioning in cancer patients. Figure 2 shows a forest plot of the ES of WCC on somatic functioning in cancer patients.

#### 3.3.2 Depression

**FIGURE 3:**
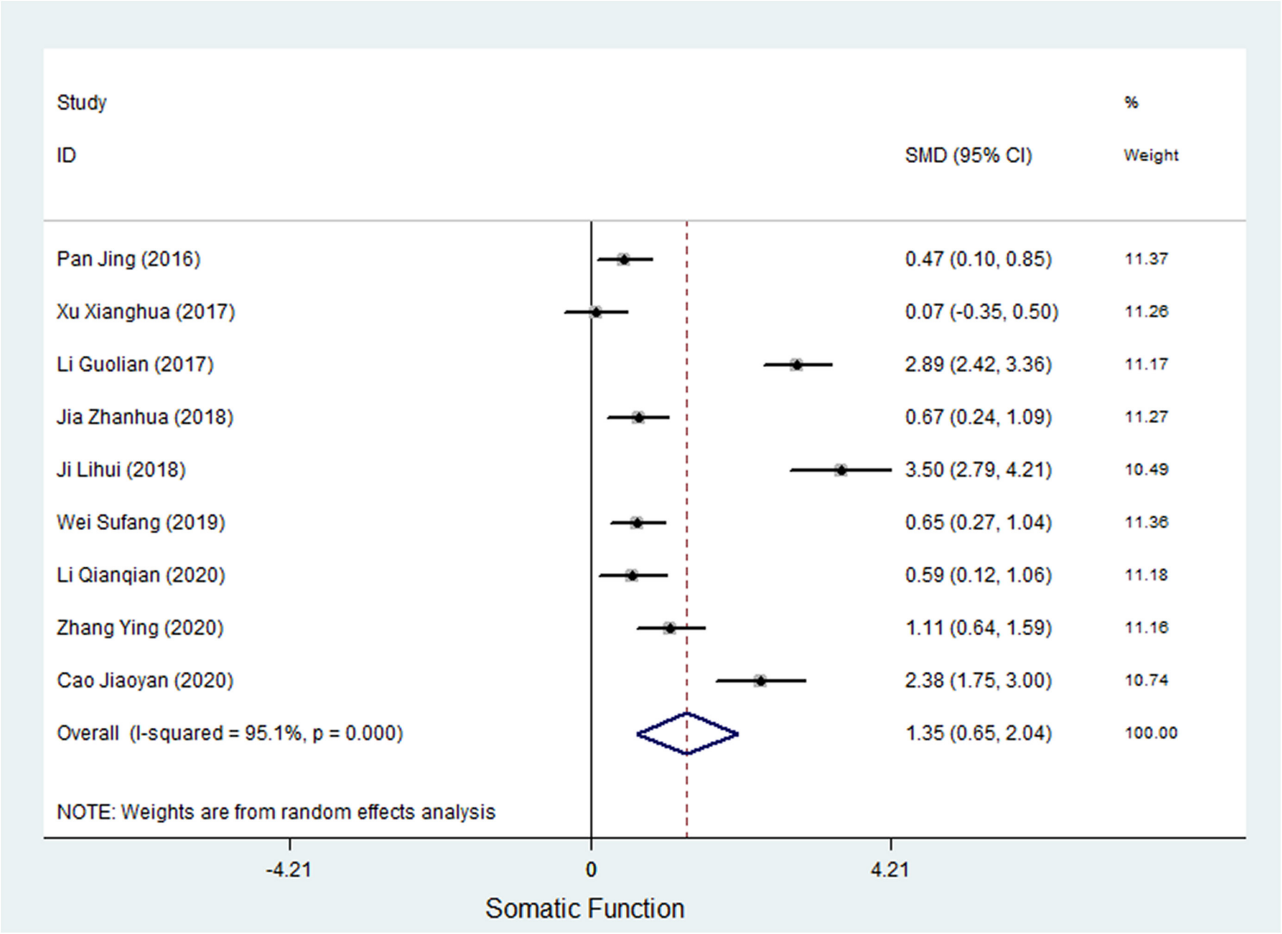
A forest plot of the ES of WCC on Depression in cancer patients.

Eight studies measured depressive symptoms in cancer patients (Chen & Wang, 2016; Dongfang et al., 2019; Min et al., 2018; Qianqian, 2020; Xianghua et al., 2017; Xiaoyu & Kuiling, 2017; Yu, 2017; Zhanhua, 2018). Of the 8 studies, 6 studies used the Self‐rating Depression Scale (SDS), and 2 studies used the Hamilton Depression Rating Scale (HAMD) to evaluate depression symptoms in cancer patients. Meta‐analysis showed a heterogeneity test result of *I* ^2^ = 63.2%, and the SMD of the combined ES was −1.104 (95% CI, −1.373 to −0.836; *p* < 0.000), suggesting that WCC can improve depression symptoms in cancer patients. Figure 3 shows a forest plot of the ES of the WCC on depression in cancer patients.

#### 3.3.3 Anxiety

**FIGURE 4:**
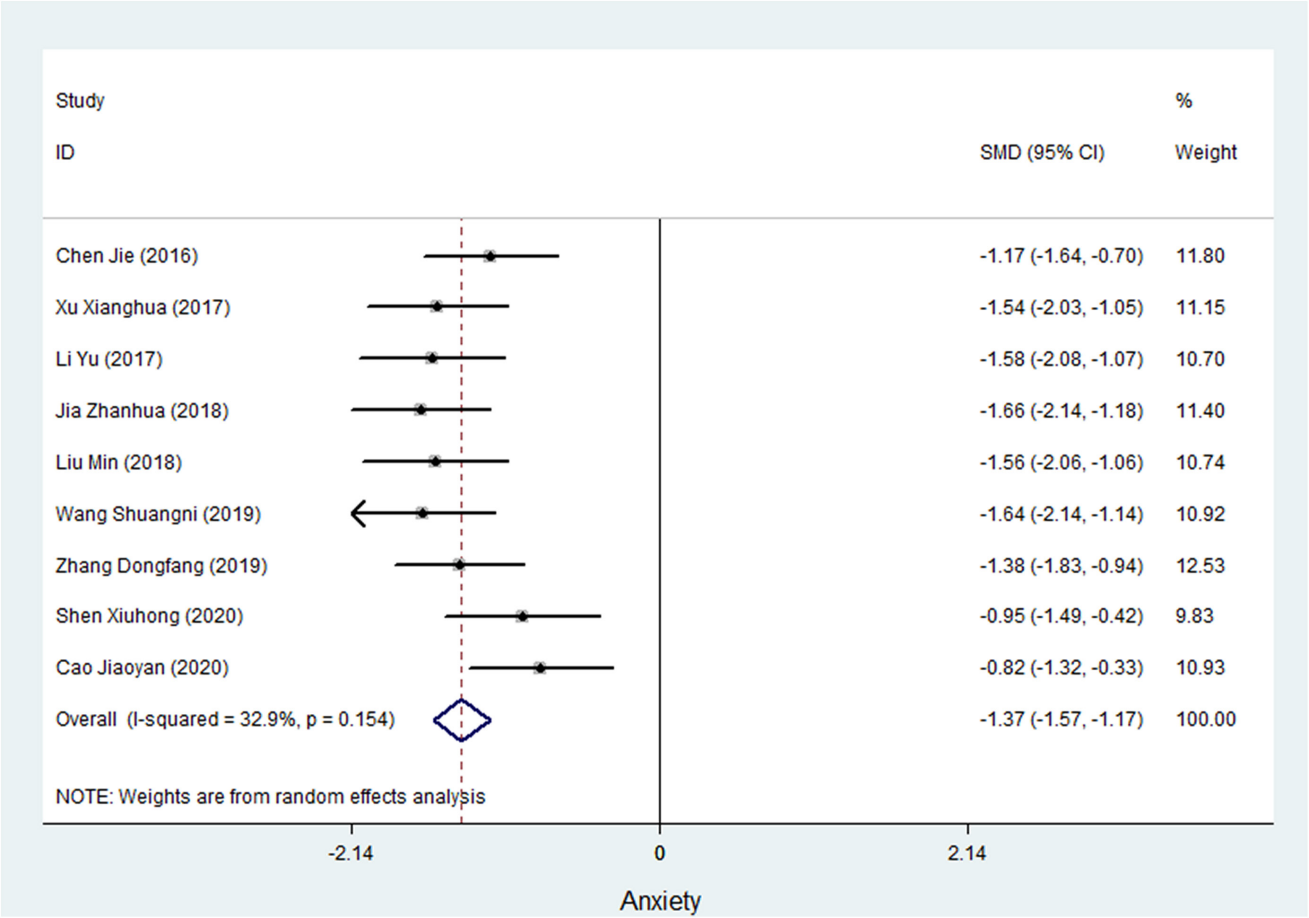
A forest plot of the ES of WCC on Anxiety in cancer patients.

Nine studies measured anxiety symptoms in cancer patients (Chen & Wang, 2016; Dongfang et al., 2019; Jiaoyan et al., 2020; Min et al., 2018; Shuangni et al., 2019; Xianghua et al., 2017; Xiuhong et al., 2020; Yu, 2017; Zhanhua, 2018). Of the 9 studies, 7 studies used the Self‐rating Anxiety Scale (SAS), and 2 studies used the Hamilton Anxiety Rating Scale (HAMA) to evaluate anxiety in cancer patients. Meta‐analysis results showed that the test for heterogeneity was *I* ^2^ = 32.6%, and the SMD of the combined ES using a fixed‐effects model was −1.372 (95% CI, −1.535 to −1.209; *p* < 0.001), suggesting that WCC could improve the anxiety symptoms of cancer patients. Figure 4 shows a forest plot of the ES of the WCC on anxiety in cancer patients.

#### 3.3.4 Social function

**FIGURE 5:**
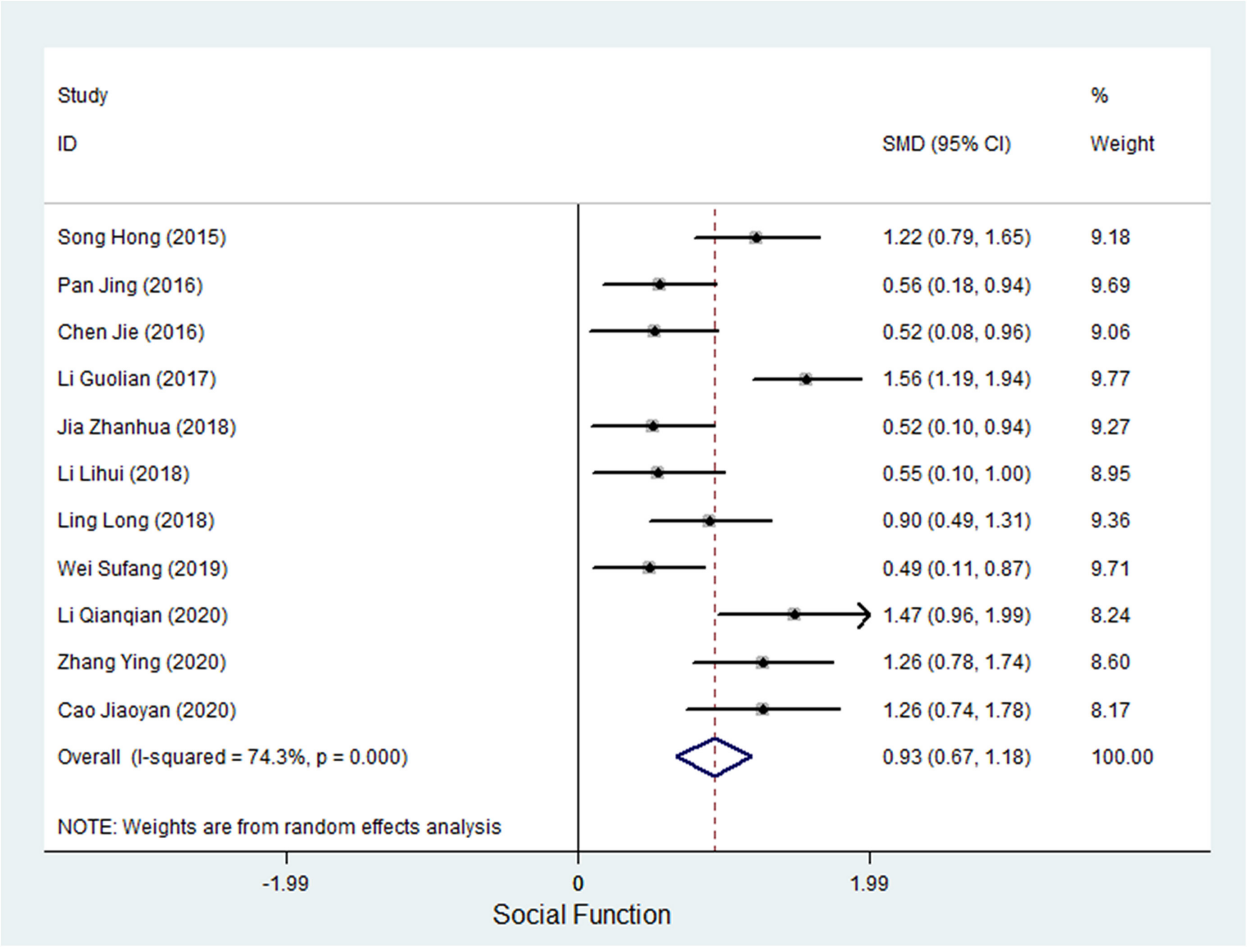
A forest plot of the ES of WCC on Social Function in cancer patients.

Eleven studies measured the social function of cancer patients (Guolian, 2017; Hong, 2015; Jiaoyan et al., 2020; Chne Jie et al, 2016; Jing & Yajuan, 2016; Lihui et al., 2018; Long et al., 2018; Qianqian, 2020; Sufang & Xiaoya, 2019; Ying & Fang, 2020; Zhanhua, 2018). Of the 11 studies, 4 studies used the EQRTC QLQ‐C30 to evaluate the social function of cancer patients, 3 studies used the FACT‐B, 1 study used the SF‐36 (to evaluate social competence), 1 study used the COH‐QOL‐OQ, 1 study used the Psychosocial Adjustment to Illness Scale (PAIS‐SR), and 1 study used the Herth Hope Index (HHI). Meta‐analysis results showed that the heterogeneity test result was *I* ^2^ = 74.3%, and the SMD of the combined ES using a random‐effects model was 0.928 (95% CI, 0.671–1.185; *p* < 0.001), suggesting that WCC can improve the social competence of cancer patients. Figure 5 shows a forest plot of the ES of the WCC on the social functioning of cancer patients.

#### 3.3.5 Cognitive function

**FIGURE 6:**
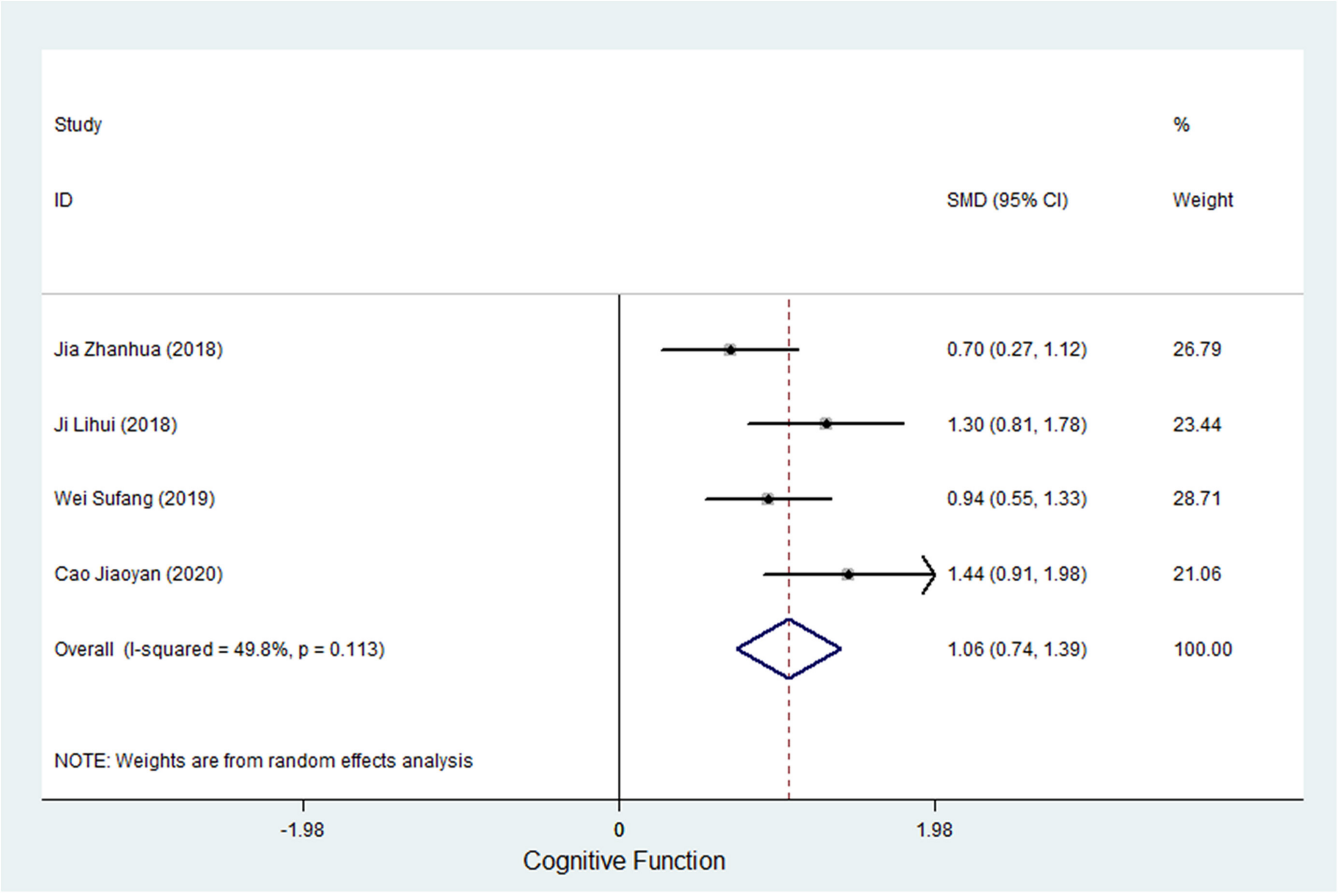
A forest plot of the ES of WCC on Cognitive Function in cancer patients.

Four studies measured the cognitive function of cancer patients (Dongfang et al., 2019; Jiaoyan et al., 2020; Lihui et al., 2018; Long et al., 2018), and all four studies used the EQRTC QLQ‐C30 to evaluate the social cognitive abilities of cancer patients. Meta‐analysis results showed that the heterogeneous test outcome was *I* ^2^ = 49.8%, and the SMD of the combined ES using a fixed‐effects model was 1.038 (95% CI, 0.813–1.264; *p* < 0.001), suggesting that WCC can effectively improve the cognitive ability of cancer patients. Figure 6 shows a forest plot of the ES of WCCs on the cognitive function of cancer patients.

### 3.4 Heterogeneity and sensitivity analyses

Meta‐analysis results showed high heterogeneity among studies evaluating WCC for improving somatic functioning, depression, and social functioning in cancer patients, while showing homogeneous results in the other two areas. Sensitivity analyses were performed on the results of all meta‐analyses and showed that all estimates were within the lower and upper CI limits. For the results of the sensitivity analyses, see Appendix S1.

### 3.5 Publication bias

In the 5 meta‐analyses, a large publication bias was found with the Begg test for the somatic function outcome, with the exception of the outcome indicator of physical function, which was found to be robust when recalculated without the safety factor. No significant publication bias was found with the Begg test (*p* > 0.05) or NFS values (NFail‐safe > criterion) for other outcome indicators. The results of the heterogeneity tests and publication bias assessments are shown in Table 2.

**TABLE 2:** Results of heterogeneity and publications bias analyses.

Group	Sample size		Heterogeneity				Effect size			Publication bias				
	*K*	*N*	*χ* ^*2*^	df	*p*	*I* ^2^, %	SMD	95% CI	*p*	*N*_fs0.05_	Criterion	Begg‐test		
												*Z*	*p*_Begg_	Model
Somatic function	9	839	162.61	8	<0.001	95.1	1.348	0.653–2.043	<0.001	1313.955959	55	2.19	0.029	REM
Depression	8	681	19.01	7	0.008	63.2	−1.104	−1.373 to −0.836	<0.001	622.3657074	50	1.61	0.108	REM
Anxiety	9	725	11.93	8	0.154	32.9	−1.372	−1.535 to −1.209	<0.001	1141.342263	55	−0.10	1.000	FEM
Social function	11	1038	38.96	10	<0.001	74.3	0.928	0.671–1.185	<0.001	915.7869426	65	1.09	0.276	REM
Cognize	4	549	5.97	3	0.113	49.8	1.038	0.813–1.264	<0.001	143.9924421	30	1.02	0.308	FEM

Abbreviations: CI, confidence interval; FEM, fixed‐effects model; SMD, standardized mean difference.

### 3.6 Subgroup analysis

Subgroup meta‐analyses were performed to better understand the effects of different interventions within the WCC interventions for cancer patients (Table 3). In a five‐item meta‐analysis, it was found that different WCC interventions improved somatic functioning, anxiety, depression, social functioning, and cognitive functioning in cancer patients (*p* < 0.05). Regarding physical function, SMD_1_ > SMD_3_ > SMD_2_ among the three interventions, which indicated that the mixed interventions resulted in better improvements in physical function in cancer patients. Regarding depression, SMD_1_ > SMD_3_ > SMD_2_ among the three interventions, which indicated that the mixed interventions resulted in better improvements in depression in cancer patients. Regarding anxiety, among the three interventions, SMD_3_ > SMD_1_ > SMD_2_, which indicated that the mixed interventions resulted in better improvements in anxiety in cancer patients. Regarding social function, among the three interventions, SMD_1_ > SMD_3_ > SMD_2_, which indicated that the mixed interventions resulted in better improvements in the social function of cancer patients. Regarding cognitive function, among the three interventions, SMD_3_ > SMD_1_ > SMD_2_, which indicated that the mixed interventions resulted in better improvements in cognitive function in patients with cancer.

**TABLE 3:** Subgroup analysis of WeChat‐based continuous care intervention on cancer patients.

	1. Mixed interventions				2. Mixed interventions and psychological interventions				3. Mixed interventions and rehabilitation exercises			
	Effect size				Effect size				Effect size			
	SMD_1_	95% CI	*p*	*I* ^2^, %	SMD_2_	95% CI	*p*	*I* ^2^, %	SMD_3_	95% CI	*p*	*I* ^2^, %
Depression	−0.986	−1.411 to −0.562	<0.001	—	−1.151	−1.569 to −0.733	<0.001	70.6	−1.104	−1.667 to −0.598	<0.001	76.4
Anxiety	−1.348	−1.630 to −1.066	<0.001	42.2	−1.449	−1.726 to −1.173	<0.001	10.5	−1.314	−1.603 to −1.025	<0.001	65.2
Social function	1.069	0.640–1.498	<0.001	76.7	0.697	0.368–1.026	<0.001	57.6	1.054	0.419–1.689	0.001	—
Cognize	1.298	0.811–1.784	<0.001	—	0.698	0.272–1.124	0.001	—	1.157	0.669–1.645	<0.001	—
Somatic Function	3.135	2.553–3.718	<0.001	49.1	0.573	0.172–0.974	0.005	72.0	1.180	0.203–2.157	0.018	91.9

*Note*: Effect size calculated by random‐effects model.

Abbreviations: CI, confidence interval; SMD, standardized mean difference.

## 4 DISCUSSION

The reliability of the results of this meta‐analysis was closely related to the quality of the literature included in the original study. Of the 18 studies, 12 studies (Dongfang et al., 2019; Hong, 2015; Jiaoyan et al., 2020; Jing & Yajuan, 2016; Long et al., 2018; Min et al., 2018; Shuangni et al., 2019; Sufang & Xiaoya, 2019; Xianghua et al., 2017; Xiaoyu & Kuiling, 2017; Xiuhong et al., 2020; Ying & Fang, 2020) randomly assigned subjects to groups, 8 studies (Dongfang et al., 2019; Hong, 2015; Long et al., 2018; Sufang & Xiaoya, 2019; Xianghua et al., 2017; Xiuhong et al., 2020; Ying & Fang, 2020; Zhanhua, 2018) implemented allocation concealment, and none of the 18 studies mentioned blinding. Since WCC interventions are out‐of‐hospital follow‐up interventions, it is difficult to blind the interventionists, so it is inevitable that there will be some bias. However, a blinding method could have been applied to the measurement of outcome indicators to avoid bias in the measurement results.

Cancer is a malignant disease involving abnormal cell growth (Maman & Witz, 2018), which leads to millions of new cases and deaths every year. Therefore, the implementation of a continuous follow‐up strategy and reasonable out‐of‐hospital guidance is of great significance to improve the quality of life and survival rate of cancer patients. In previous studies, the continuity of care interventions for cancer patients have focused on psychological interventions (Muzi, 2016), relaxation training (Jing et al., 2019), exercise therapy (Hua, 2017), and systematic nursing interventions (Chuanlin et al., 2014; Maimaiti et al., 2013). In this study, we focused on the effects of applying WCC interventions in cancer patients. Due to different intervention methods and measurement methods of the outcome indicators adopted by the different researchers, this paper selected five outcomes, including somatic function, depression, anxiety, social function and cognitive function, from 18 RCTs for meta‐analysis.

Meta‐analysis results showed that the WCC intervention was effective in improving somatic function in cancer patients, which was inconsistent with the results of previous studies (Wang et al., 2017). A possible reason for this was the high heterogeneity of the results in the meta‐analysis for somatic function in this study (*I* ^2^ = 95.1%). Although caregivers were able to remind patients to take their medications through the WeChat platform, clarify the side effects of relevant medications, and reinforce their compliance behaviours (Xiaowei & Hong, 2020), cancer is an organic disease, and more rigorous and large sample size intervention studies are needed in the future to explore the effectiveness of WeChat intervention programmes for improving somatic function in cancer patients.

In terms of mental health, compared to conventional nursing interventions, WCC interventions can improve patients' anxiety and depression, which was similar to the findings of Yingying et al. WCC interventions differ from conventional discharge follow‐up in that WeChat is currently the most used social software in China, and health care workers can communicate with cancer patients through WeChat groups or use the WeChat video call function to monitor the mental status of cancer patients; this allows for identifying patients' psychological problems, providing timely guidance, and helping cancer patients to actively and effectively improve their negative emotions.

In terms of social functioning, WCC interventions can be effective in improving the social function of cancer patients. Reintegration of cancer patients into society after treatment is an essential part of the recovery process (Syrjala et al., 2010); therefore, cancer patients can strengthen communication with other patients through WeChat groups, and this experience of sharing and exchanging among patients with better prognoses can make patients feel supported and inspired, which increases the confidence of other patients to overcome the disease and is conducive to faster reintegration of patients into society and further improving prognosis.

In terms of cognitive function, WCC interventions can improve cognitive function. Studies have shown that cancer patients' daily cognitive function gradually decreases after the end of treatment (Von Ah & Crouch, 2021), and some even experience a decline in memory, processing speed, attention and executive ability (Ono et al., 2015), which requires healthcare professionals to provide cancer patients with specific programs to improve memory and enhance coping cognitive function. On the other hand, health care workers can monitor their rehabilitation exercises, diet and medication intake and prompt patients to review and participate in hospital activities through the WeChat platform.

The results of the subgroup analyses suggested that the mixed interventions produced greater effects on improving somatic functioning, depressed mood, and social functioning in cancer patients and that the mixed interventions combined with rehabilitation exercises produced greater effects on improving anxiety and cognitive functioning in cancer patients. Mixed interventions incorporated rehabilitation interventions that produced greater effects on improving somatic functioning, anxiety, depressed mood and social functioning, with smaller effects on cognitive functioning. One possible reason for this result is the small number of trials considered in the meta‐analysis, which inevitably reduces the validity of these analyses. Therefore, more experimental studies in related areas are needed in future research to better understand the effects of different interventions.

Recording the detailed usage of WeChat is very valuable for better drawing on existing experiences in future interventions. However, in the analysis of 18 RCTs included in this study, we found that none of the studies gave an introduction on how to record the usage of WeChat. This is detrimental to the implementation of WCC interventions in the future. We also call for more open reporting of detailed intervention processes in future studies.

Partial heterogeneity was found in this study, and in the meta‐analysis, the large heterogeneity may be due to dissimilarities between studies, which mainly included differences in study population, study design, interventions, and measurement outcomes. Since heterogeneity was unavoidable, random‐effects models were used to address possible variation among studies. Sensitivity analysis of the results of the meta‐analysis with high heterogeneity among the studies was shown to be robust. Regarding publication bias, funnel plots were not chosen as an evaluation indicator due to the small number of studies included in this paper. Here, a combination of Begg's tests and NFS was used to evaluate publication bias, and the results showed that there was no significant publication bias in this study.

There are some limitations in this study. First, the literature search was limited to articles in Chinese and English, which may have led to the literature selection bias. Second, studies with negative outcomes are often difficult to publish, which may lead to publication bias. Finally, given the study limitations in the included literature, only four outcome indicators were selected in this study to evaluate the effectiveness of WCC in cancer patients; in subsequent RCTs, we suggest that more outcome indicators should be examined to evaluate the effectiveness of WCC interventions. Despite the aforementioned shortcomings, the results of this study suggest the positive value of WCC interventions in improving anxiety, depressed mood, social functioning, and cognitive function in cancer patients.

## 5 CONCLUSIONS

In summary, this study combined and analysed the currently available literature to more systematically evaluate the results of multiple studies. The results of this study showed that WCC interventions were effective in improving depression, anxiety, social functioning, and cognition in cancer patients with a positive effect compared with conventional out‐of‐hospital follow‐up. Given the small number of studies and their methodological limitations, these results should be regarded as preliminary and interpreted with caution.

## CONFLICT OF INTEREST STATEMENT

There are no conflicts of interest.

## ETHICS STATEMENT

This study was approved by the Ethics Committee of the Bengbu Medical College (2017054).

## REGISTRATION AND PROTOCOL

This review was not registered.

## Supporting information


Appendix S1

## Data Availability

All datas is from the quoted articles listed in the reference list.
