# Supplementary material for: Effect of WeChat‐based continuous care intervention on the somatic function, depression, anxiety, social function and cognitive function for cancer patients: Meta‐analysis of 18 RCTs
Source: Nurs Open. 2023 Jun 26;10(9):6045–57. doi: 10.1002/nop2.1916 (PMC10416022; doi:10.1002/nop2.1916)
Supplement: Supplementary file 1 — Appendix S1 [file NOP2-10-6045-s001.docx]

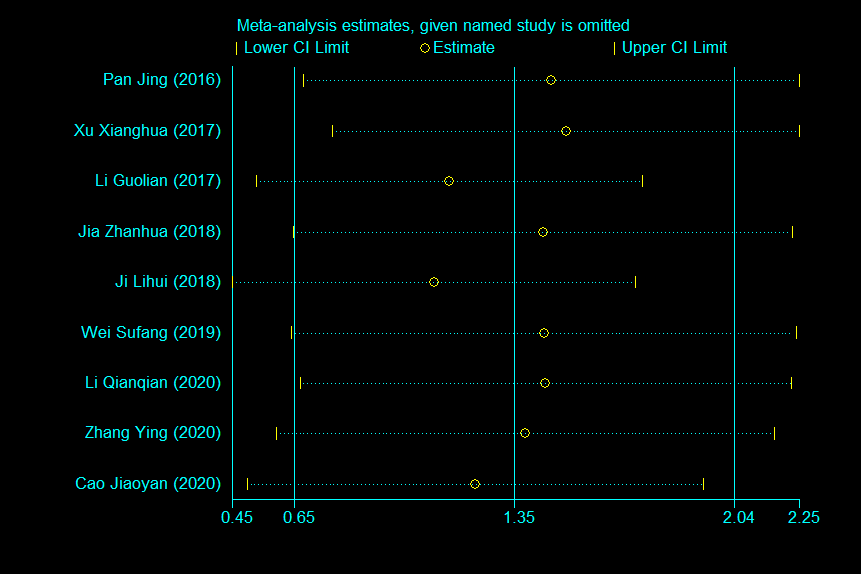


Figure A. Somatic Function


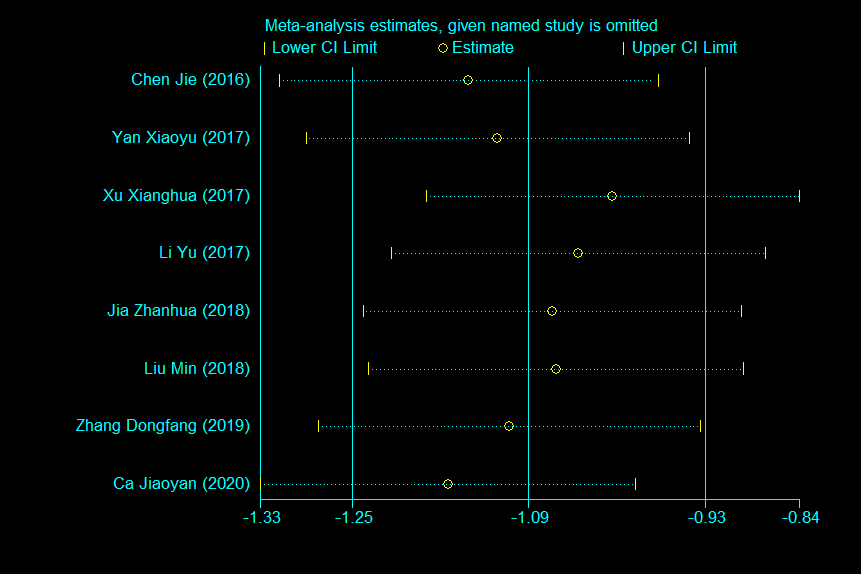


Figure B. Depression


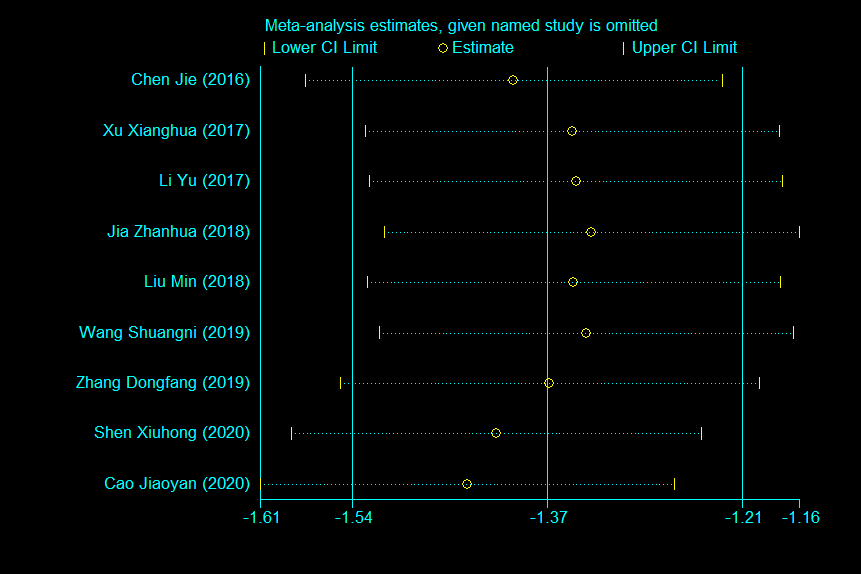


Figure C. Anxiety


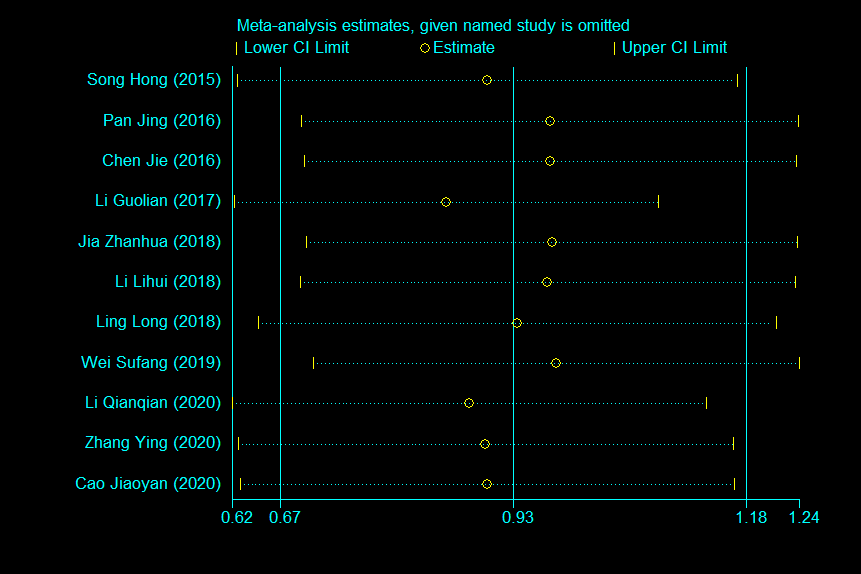


Figure D. Social Function


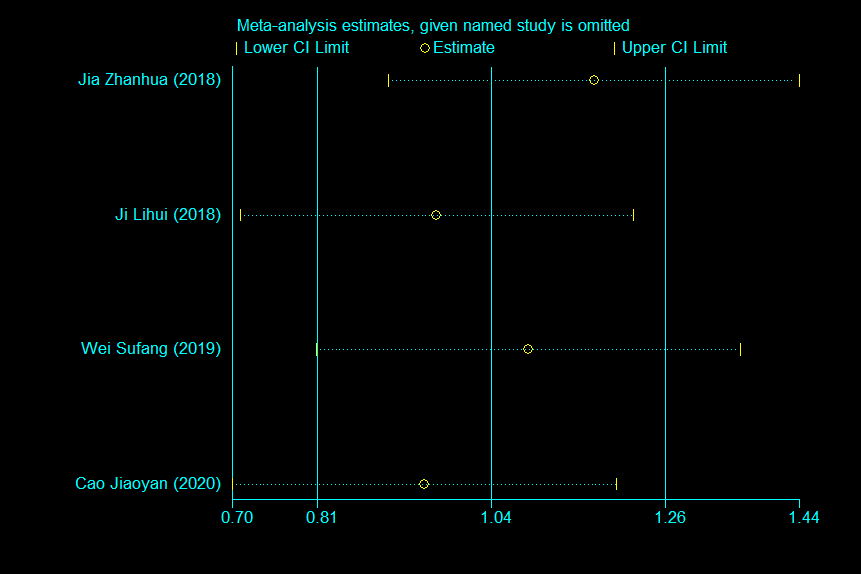


Figure E. Cognitive Function
